# Evaluation of Patients’ Preferences for Skin Grafting in Plastic-Surgical Defect Coverage

**DOI:** 10.29252/wjps.9.3.259

**Published:** 2020-09

**Authors:** Lukas Fabian Busch, Seyed Arash Alawi

**Keywords:** Split-thickness skin graft, Full-thickness skin graft, Scalp skin donor site, Graft localization

## Abstract

**BACKGROUND:**

Grafting split-thickness (STSGs) and full-thickness skin grafts (FTSGs) are common techniques to replace missing skin and to restore the skin barrier in burn, trauma and remaining skin defects after tumor resections. The defect coverage with skin grafts offer many advantages, but also disadvantages such as donor site morbidity like possible sensory disturbances, scarring, risk of infection, contour changes and pigment disorders. We aimed to assess the preferred distribution of donor site for STSGs and FTSGs in patient’s skin grafting for plastic-surgical defect coverage.

**METHODS:**

Patients and their accompany persons referred to the Department of Plastic Surgery were interviewed for defect coverage with STSGs or FTSGs, the preference in donor site was investigated and the detailed advantages and disadvantages were clarified.

**RESULTS:**

We evaluated 85 participants (male=43, female=42) with a median age of 42 years (mean=46 years). The definition of the donor site (n=188 markings) was mainly based on the physicians recommendation (32.98%), mobility (23.40%), aesthetic results (22.34%) and pain (21.28%). Feared complications (n=152) were mainly wound healing disorders (32.24%), circulation disorders (28.29%), scars (20.39%) and bleeding risks (19.08%)**.** Among all participants, 79 split-skin graft preferences were specified, while 32% favored the scalp as a donor site, as well as 29% the frontal part of the left thigh and 10% the frontal part of the right thigh.

**CONCLUSION:**

There were preferred anatomical donor sites for skin grafting. Nevertheless, in conscious patients, the donor site has to be selected in a consent talk and joint approval, preoperatively. The options of taking STSGs from the occipital region with all its advantages should be discussed intensively as it is an attractive graft donor site.

## INTRODUCTION

Autologous skin grafts, like split- or full-thickness skin grafts, in Meek and Mesh techniques are common techniques to reconstruct the skin and to restore the skin barrier in acute or chronic wounds.^1^^,^^2^ In regard to the reconstructive ladder, it is one of the first plastic surgical procedures to be considered.^3^ Skin grafts can be divided into full- or split-thickness epidermal skin grafts. Skin grafts can generally be divided into autografts, homograft and xenograft. Skin is a highly immunoreactive tissue and only autografts will heal in the transplanted area.^4^

The benchmark is to achieve complete wound healing in a very short time with minimal graft donor site morbidity. By using autologous skin grafts, an effective option to cover skin defects exists. In addition, skin grafts also seem to have a local regenerative effect.^5^ Already 3000 years ago, split-thickness skin grafts (STSG) were used in India for tissue reconstruction after punishments with tissue damage.^6^ In Europe mainly Gaspare Tagliacozzi of Bologna (1546–1599) developed the technique and published his work “De Curtorum Chirurgia per Insitionem (Surgery of the Mutilated by Grafting)” in 1597. In course of modern surgery, it was revived about 1817 in Germany for reconstruction of nasal defects.^7^

Also in Switzerland Jacques-Louis Reverdin (1842–1929) developed his approach for skin grafting by using skin pieces of small, full-thickness skin grafts (FTSG). Regular donor sites were the lateral thigh and buttocks. Also in children, occipital donor sites are a common graft donor site for STSGs and were demonstrated with good outcomes regarding donor site complications and graft healing.^8^^-^^10^ By transplantation of STSGs, a rapid defect healing and fast donor site healing were desired. Even if there are no studies comparing all possible donor sites regarding complications and skin take rate in a randomized controlled trial, the positive features of occipital donor sites were previously demonstrated.^10^

In terms of complications like bleeding, pruritus and scarring, occipital donor site may have advantages compared to a femoral donor site.^11^ Also based on frequent hair follicles as well as more adnexal structures, a high concentration of epidermal stem cells are available for regeneration in the donor.^12^^,^^13^ Regarding occipital donor site, good results were illustrated regarding graft take and healing rates. However, skin grafts are commonly taken from the thigh, even if good results were demonstrated for occipital donor sites.^14^

In addition, prospective studies showed advantages of occipital donor site compared to the thigh. Particularly, it is a significant better reepithelization, better cosmetic outcome, better results on Vancouver Scar Scale and less pain.^14^ This graft donor site is mostly established in children and burn patients, but should also be offered to further patient collectives.^10^^,^^15^ The use of STSG and FTSG can be considered in burn patients, in reconstructive surgery, and in hand surgery but also in the therapy of chronic wounds.^16^

Additionally the use of STSG can be combined with negative pressure wound therapy (NPWT) as an additional preparation of the wound bed before grafting, but also to enhance the healing process of the STSG postoperatively.^16^ Investigations showed that the use of NPWT can enhance the graft take, reduce complications like seroma and hematoma too, but also enhance the quality of healing.^17^^,^^18^ The use of STSG in chronic diabetic wounds also showed an enhanced healing process in comparison to conservative therapy with paraffin gauze and iodine dressing.^19^

According to experiences mainly the consent talk is influencing the donor site position. In the context of the presentation of the advantages and disadvantages of STSG, as well as FTSG donor sites, an evaluation of the preferred donor site is necessary. By explaining the patient all possible donor site areas and their associated characteristics, we hypothesize that occipital donor sites are preferred by the patients based on the good clinical results and advantages. 

## MATERIALS AND METHODS

**Fig. 1:**
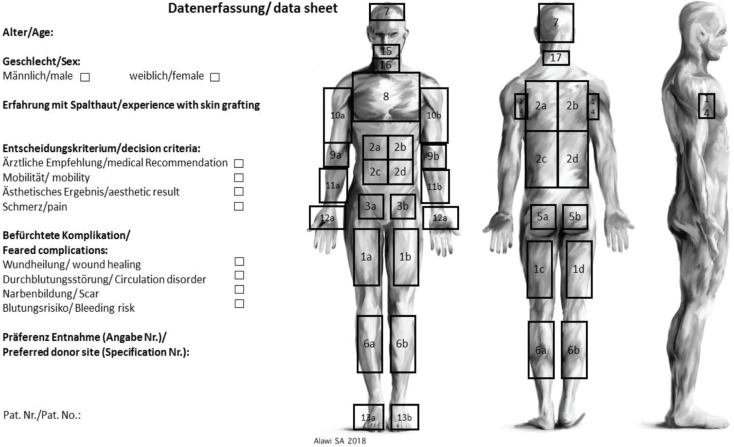
Data collection sheet with possible marking positions. Multiple choice selection for skin graft preferences was possible

We questioned all patients and their accompanied persons referring to our Department of Plastic Surgery in order to clarify the procedure with potential defect coverage including STSGs and FTSGs and to inquire the preferences of different donor sites too. The detailed advantages and disadvantages were clarified. The preferred donor sites for STSG and FTSG donor sites were marked on a data sheet (Figure 1). We collected data such as age, gender, experience with skin grafting, skin color (evaluation of Fitzpatrick skin type) and preferred donor site for STSGs and FTSGs. Multiple-choice selection regarding skin graft preferences was possible. Research was conducted in accordance with the 1964 Helsinki Declaration. The ethical committee from Hannover Medical School (No. 7474) approved this study. Patient informed consent was taken for the presented pictures as well as for the participation in the survey. 

## RESULTS

**Fig. 2:**
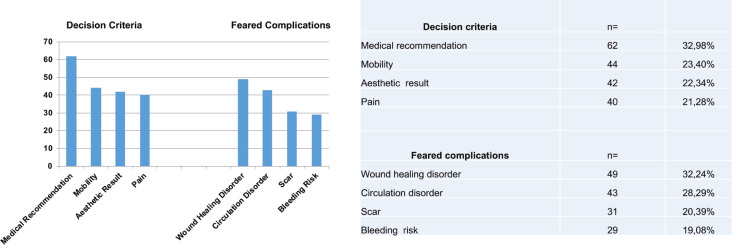
Mainly the Fitzpatrick skin type II (n=36, 42.3%) and III (n=37, 43.5%) were represented (Type I=3, Type IV=9, Type V=3, Type VI=0). The decision making regarding the donor site (n=188 markings) was mainly based on the physicians recommendation (n=62, 32.98%), mobility (n=44, 23.40%), aesthetic result (n=42, 22.34%) and pain (n=40, 21.28%).

**Fig. 3:**
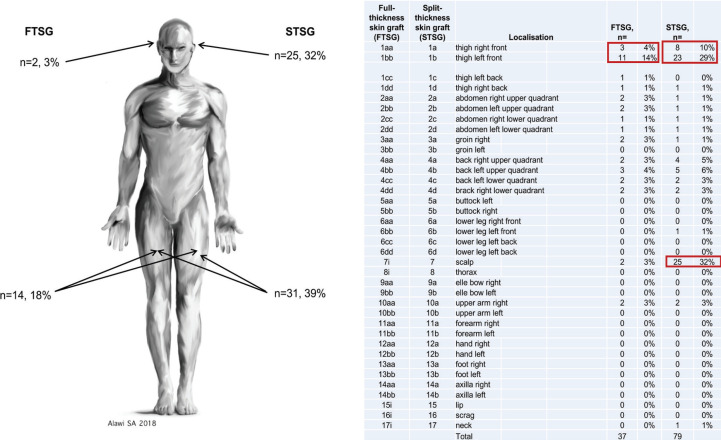
Feared complications (n=152) were mainly wound healing disorders (n=49, 32.24%), circulation disorder (n=43, 28.29%), scars (n=31, 20.39%) and bleeding risks (n=29, 19.08%).

We evaluated 85 participants (male=43 and female=42) with a median age of 42 years (Mean=46 years). From those, 11.7% had experienced STSG. About 2.4% had experience with full-thickness grafts. Mainly, the Fritzpatrick skin type II (n=36, 42.3%) and III (n=37, 43.5%) were represented (Type I=3, Type IV=9, Type V=3, Type VI=0). The definition of skin donor sites (n=188 markings) was mainly based on the physicians’ recommendation (n=62, 32.98%), mobility (n=44, 23.40%), aesthetic results (n=42, 22.34%) and pain (n=40, 21.28%, Figure 2). Feared complications (n=152) were mainly wound healing disorders (n=49, 32.24%), circulation disorders (n=43, 28.29%), scars (n=31, 20.39%) and bleeding risks n=29, 19.08%, Figure 2). Of 85 participants, 79 STSG donor site preferences were specified. Of the given STSG preferences, 32% (n=25) preferred the scalp, as well as 29% (n=23), the frontal left thigh and 10% (n=8), the frontal right thigh. For FTSG the ventral left thigh was preferred in 35% despite being informed that a full skin removal would be atypical in this area (Figure 3). 

**Fig. 4:**
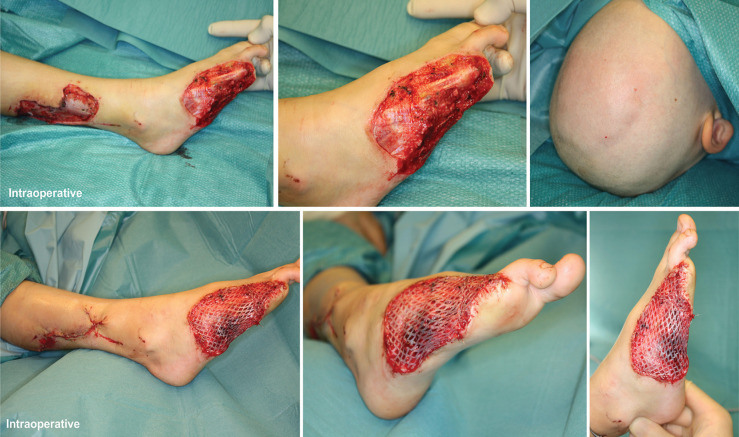
A 6-year-old boy who has a skin defect after a lawn mower injury. At the first presentation in the emergency department, an amputation of the metatarsal V bone down to the base, an amputation of the metatarsal bone DIV at the diaphysis, as well as a separation of the third toe in the MCP joint were shown. In addition, a soft tissue defect was present in the area of ​​the lateral lower leg. A replantation of the amputated tissue was not possible due to massive tissue damage. After previous debridement and wound conditioning by means of vacuum therapy, the closure of the defect in the area of ​​the lower leg was carried out with local large-area advancement flap. A local muscle advancement flap of ​​the foot was performed based on the abductor digiti minimi, flexor digiti minimi brevis and interosseus dorsalis III and IV. To cover the skin defect, we transplanted split-thickness skin graft from the head of the child

Figure 4 shows a 6-year-old boy who has a skin defect after a lawn mower injury. At the first presentation in the emergency department, an amputation of the metatarsal bone V down to the base, an amputation of the metatarsal bone DIV at the diaphysis, as well as an amputation of the DIII at the metacarpophalangeal joints (MCP) were shown. In addition, a soft tissue defect was present in the area of the lateral lower leg. A replantation of the amputated tissue was not possible due to massive tissue damage. After previous debridement and wound conditioning by means of vacuum therapy, the closure of the defect in the area of the lower leg was carried out with a local large-area advancement flap. A local muscle flap was performed with the abductor digiti minimi, flexor digiti minimi brevis, and interosseus dorsalis III and IV. To cover the skin defect, we transplanted STSG from the occipital region. 

**Fig. 5:**
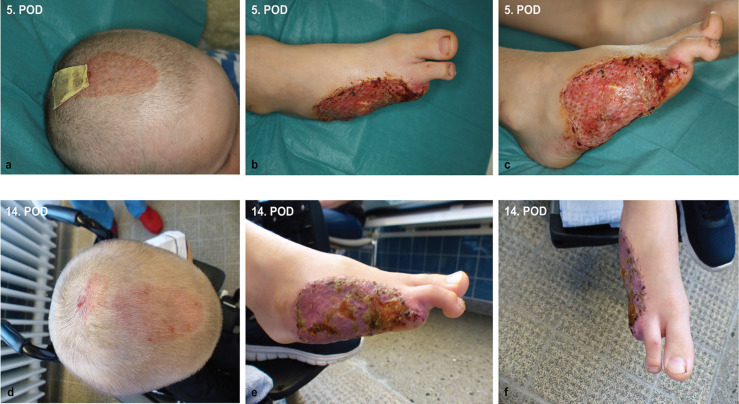
The split-thickness skin graft on the head as well as the transplanted split-thickness skin graft on the foot showed almost complete healing after 5 days **(a-c).** No complications were noted of the donor site with a sufficient healing of the scalp as well as in the area of the transplanted split-thickness skin graft **(d-f).**

Figure 5 demonstrates the split-thickness skin donor site on the head as well as the transplanted split-thickness skin graft on the foot, which showed almost complete healing after 5 days. No complications was noted of the donor site with a sufficient healing of the scalp as well as in the area of the transplanted split-thickness skin graft.

## DISCUSSION

Based on our study, the decision of skin donor sites was mainly based on the physicians’ recommendation. Simultaneously, most feared complications were wound healing and circulation disorders (Figure 2). Of the given STSG preferences, in 32% (n=25) the scalp was selected as a preferred donor site, as well as in 29% frontal left thigh and in 10% the frontal right thigh. For FTSG, the left thigh would be indicated ventrally despite being informed that a full skin removal would be atypical in this area (Figure 3). Skin grafting is a commonly used surgical procedure for recovering skin defects. Mainly grafts are taken from the thigh, the abdomen and the buttock based on the simple access and the abundant harvesting area. The indication for the use of STSG or FTSG has to be considered individually. Beside the conservative treatment of wound healing, it is an effective and fast solution for skin coverage. Grafting of skin is generally an easy and standardized surgical procedure. 

But beside the advantages of split- or full-thickness skin grafts, complications like scarring, hypertrophic scarring, superinfection, pain while moving, bleeding and secretion should be considered. However, the scalp as a donor site is very reliable and complication rates for STSG from the scalp are very low as described in previous studies.^20^ A complication rate between 1.8% and 6% was described based on complications like folliculitis, alopecia and bleeding. Hypertrophic scarring as well as keloids at the donor site were not present. Some patients may develop folliculitis and alopecia at the scalp donor site and this is the most common complication.^21^

Permanent alopecia either of the entire donor’s site or punctually is the most feared donor site complication. The reported incidences vary between 1%^22^ to 13% in unburned scalp.^23^ Too-deep harvesting^24^ as well as quick reharvesting enhances the incidence of alopecia or microalopecia and folliculitis.^10^ A special emphasis has to be taken on the use of the dermatome as studies showed a significant variation of STSG thickness of 0.12 mm to 0.42 mm based on the pressure and individual handling of the device.^25^

In order to reconstruct defects, we have to describe all possible graft donor sites to the patient. Figure 2 shows the distribution of favored skin grafting donor sites. By describing possible complications, occipital donor sites seem to be favored, even if a hair-cut is needed. It was shown that an occipital skin regeneration happens between 5 to 8 days with the superiority of occipital skin grafting independent of age.^14^ The advantages of occipital donor sites initially described by Crawford in 1964 were also confirmed.^26^

The healing duration seems to be shorter than the thigh.^11^^,^^14^ Mainly the excellent vascularization as well as stem cell concentration in the hair shafts may be the reason for good regeneration.^13^^,^^27^ In addition, we should consider the lower pain level through less mobilization in the occipital region, which was confirmed before.^14^ Finally, the harvesting frequency can be higher based on the epithelialization frequency and duration. The only limitation was the maximum area of graft takes with about an equivalent of 7% of total body skin surface, which can be harvested multiple times. In adults, less total body skin surface can be covered based on the lower proportion of scalp to the further body skin surface. The superiority of scalp STSG donor site in regard of the healing process and possibility of repeated grafting was previously described.^22^

When using STSG as a donor site in children to cover defects, the advantageous ratio of the scalp to the total surface of the body should be taken into account. However, the decision of donor site is dependent on the total burn surface, burn position and also dependent on the operation procedure planned. For example, if the defect coverage of the back is planned, a donor site chosen on the ventral site of the patient does not make sense as a reposition of the patient is necessary. 

Based on evaluation of optimal wound dressings for fast reepithelization of the donor site closed, open and semi-open materials were tested. Semi-opened biological skin coverage with xeroform (Sherwood Medical, St. Louis, MO) lead to a healing time of approximately 10 to 12 days.^25^^, ^^28^ The regeneration of donor sites plays an important role in the treatment of burn patients or for defect coverage, as a delay in wound healing impairs possible rehabilitation or discharge from the hospital.^11^^,^^22^

Hypopigmentation, hypertrophic scars or the rectangular shape of the donor site may be aesthetically not desirable. For the scalp as a donor site for skin grafting, a possible re-grafting was described after 5 to 7 days.^11^^,^^22^ Physiological reasons may be an excellent blood supply as well as the hair follicles and further adnexal structures containing a high amount of epidermal stem cells.^15^^,^^29^^,^^30^ Mainly, the hair follicles with stem cells in the bulge region are involved in the proliferation and migration process.^12^^,^^13^^,^^31^

Postoperative bleeding is proved to be higher in harvesting areas of the thigh, presumably caused by higher tissue movement and tissue stress. In patients with occipital donor sites, microalopecia may occur, even the risk is assessed to be low.^9^^,^^14^ This complication is occurring, if the skin graft is taken too deep or if the patient suffers from postoperative folliculitis. That should be mentioned during the consent information talk with the patient. 

## CONCLUSION

Based on our study findings, the decision of skin donor sites is mainly based on the physicians’ recommendation and all donor sites with their advantages/disadvantages and risks should be discussed intensively. Occipital skin donor site should be mentioned explicitly and should be used more frequently in reconstruction of skin defects in reconstructive surgery indications. 
